# The Time to Act Is Now: Strengthening Health Systems Research Capacity, Capability and Knowledge Translation in Remote Australia

**DOI:** 10.1111/ajr.70205

**Published:** 2026-05-14

**Authors:** Alexandra Edelman, Sam Moore, Karrina DeMasi, Donna‐Maree Stephens, Vahab Baghbanian, Chris Perry, Deborah Russell, Kalinda Griffiths, Leisa McCarthy, John Humphreys, John Wakerman

**Affiliations:** ^1^ Menzies School of Health Research Charles Darwin University Alice Springs Northern Territory Australia; ^2^ College of Medicine and Dentistry James Cook University Townsville Queensland Australia; ^3^ Central Australian Aboriginal Congress Alice Springs Northern Territory Australia; ^4^ The Kids Research Institute Australia Adelaide South Australia Australia; ^5^ DMS Research & Evaluation Consulting Darwin Northern Territory Australia; ^6^ Aboriginal Medical Services Alliance Northern Territory Alice Springs Northern Territory Australia; ^7^ Poche SA+NT College of Medicine and Public Health Flinders University Darwin Northern Territory Australia; ^8^ School of Rural Health, Faculty of Medicine, Nursing and Health Science Monash University Bendigo Victoria Australia

## Abstract

**Context:**

Communities in remote Australia face poorer health outcomes but are largely recipients of services, programs and policies that are disconnected from local experiences and priorities, with some notable exceptions. Local‐level research capacity, capability and knowledge translation that respond to community and service provider priorities are key to strengthening remote health systems.

**Aim:**

To explore two questions: (1) What is needed to strengthen health systems research capacity and capability in remote Australia? (2) How can we, as a community of remote health systems researchers, enhance knowledge translation in remote Australia to address persistent health disparities?

**Approach:**

Sixty‐five remote health systems stakeholders, including service providers, funders, researchers, and policymakers participated in a research capacity building and knowledge translation workshop in Mparntwe (Alice Springs) in 2024. They identified actions to address barriers to remote health systems research and knowledge translation. We reflect on these to highlight opportunities for policy action and learning.

**Conclusion:**

Barriers to community and service‐driven research have been recognised for decades, yet feedback from remote service providers and researchers suggests that little has changed systemically despite pockets of innovation. The time to act is now. Action must include creating funding schemes that provide for cohesive and sustained investment in remote‐based health systems research partnerships that involve industry partners—especially Aboriginal Community Controlled Health Services (ACCHSs) to contribute to Closing the Gap in health outcomes. Partnerships must respond directly to community and service provider priorities, create Aboriginal and Torres Strait Islander researcher career pathways, and leverage respective partners' institutional strengths to create learning health systems. Individual ACCHSs' experiences and context‐responsive principles for engaging with remote Aboriginal communities provide guidance and lessons for other services and researchers nation‐wide.

## 1 Introduction

September 2025 marked a pivotal milestone for rural and remote health in Australia, with rural generalism officially recognised as new medical speciality practice—the second addition to specialty practice in the country in 15 years [1]. This outcome was a culmination of decades of sustained advocacy by rural and remote professional, government and academic bodies for multifaceted health care for rural and remote patients [1]. It is one of the many examples of health care, workforce and policy innovations led from rural and remote Australia to tackle persistent remote health disadvantage, which is characterised by greater morbidity, higher avoidable mortality, and lower uptake of preventive care programs when compared with outcomes in metropolitan, regional and even larger rural centres [2].

Despite such examples of policy and practice innovation, Australians living outside of major cities are still largely passive subjects of research and recipients of services, programs and policies designed elsewhere; often in large, urban centres that can be disconnected from local experiences and priorities [3]. Place‐based research (i.e., locally driven research addressing local service and community needs) in remote Australia is still hampered by barriers to sustained research engagement [2]. These barriers include geographic distances from learning hubs, fewer professional development opportunities, limited career progression and housing unavailability [4, 5]. Additional barriers for staff working in remote health services, who are essential knowledge custodians and partners in research, include limited capacity and resources to undertake research (especially in busy and under‐staffed remote care practice environments), an absence of local health priority setting, limited understanding of research processes and potential benefits, fewer training programs targeted to skills gaps and needs, and funding models that fail to sustain research engagement [2].

The proportion of the total population who are Aboriginal and Torres Strait Islander people increases with increasing remoteness—from 2.2% in major cities to 30% in remote and very remote areas [6]. Aboriginal and Torres Strait Islander people, a third of whom live in remote Australia, remain underrepresented in research and academia [7]. Research practices deliver inconsistent outcomes for Aboriginal and Torres Strait Islander communities, with challenges including training programs that are often based exclusively on Western knowledge systems [8]. Moreover, there is an urgent need to further grow Aboriginal and Torres Strait Islander research leadership, especially in areas of higher health need [8]. Aboriginal leadership is particularly important at the local level as there is higher population transience among non‐Indigenous populations in remote areas, as reflected in excessive health workforce turnover rates [2].

To explore remote health systems research capacity and capability needs and develop an action plan, the NHMRC Centre of Research Excellence for Strengthening Health Systems in Remote Australia (CRESTRA) convened a research capacity building and knowledge translation workshop in Mparntwe (Alice Springs) in March 2024. CRESTRA is a partnership between remote researchers, service providers including Aboriginal Community Controlled Health Services (ACCHSs), ACCHSs peak bodies and policymakers. CRESTRA includes a dedicated theme on building remote health systems research capacity. Sixty‐five staff from remote health services, funding bodies, researchers and policymakers met at this workshop. Expert presenters facilitated small‐group roundtables and question‐and‐answer sessions to discuss persistent barriers and strategies to overcome them. Roundtable outcomes were then utilised to reach consensus in a whole‐group discussion and documented in a post‐workshop communique and action plan [9, 10]. In this article, we reflect on these workshop discussions to explore two questions:

1. What is needed to strengthen health systems research capacity and capability in remote Australia?
2. How can we, as a community of remote health researchers, enhance knowledge translation in remote Australia to address persistent health disparities?

In our analysis, we define *capacity* as ‘the readiness of and access to resources needed for individuals and organisations to engage’ in research and knowledge translation, and *capability* as ‘individuals' knowledge and skills’ that underpin these practices [11]. *Knowledge translation*, which we highlight as critical to link research activity with policy and practice impact, is defined as a dynamic and iterative process that involves the creation, synthesis, dissemination, exchange and application of knowledge to strengthen health systems and improve health [12].

## 2 What Is Needed to Strengthen Health Systems Research Capacity and Capability in Remote Australia?

To shape discussions about capacity and capability needs, CRESTRA researchers developed five action domains representing expert consensus among the CRESTRA Leadership Group about relevant fields of action (Table 1), namely: (1) Strengthening incentives and advocacy for Aboriginal and Torres Strait Islander community‐led research and co‐design; (2) Enabling Aboriginal and Torres Strait Islander research career pathways; (3) Improving access to research education and training; (4) Creating and leveraging partnerships; and (5) Improving research funding, administration and management [10]. These domains framed discussions at the workshop about opportunities and actions.

**TABLE 1:** Five action domains to guide future investment in remote health systems research capacity and capability building, proposed in a remote research workshop held in Mparntwe (Alice Springs) in 2024.

Action domain	Challenges	Proposed actions
1. Strengthening incentives and advocacy for Aboriginal community‐led research and co‐design	Researchers sometimes lack understanding of how to conduct place‐based, culturally responsive research that embodies co‐design principles. This can lead to disempowering research practices that fail to recognise and compensate services and communities for their time and knowledge Short‐term funding cycles, requiring casual or short fixed‐term employment contracts, can challenge recruitment and retention of community‐based researchers Recruitment processes can be inflexible and time‐consuming, lacking responsiveness to local circumstances and needs	Develop and share guidance on co‐design principles Employ embedded research brokers who understand research, are connected to the community, and who can communicate cross‐culturally Advocate for Aboriginal‐led and co‐designed health systems research in funding schemes Properly remunerate community members and services for research engagement
2. Enabling Aboriginal researcher career pathways	Discontinuity of research funding leads to job instability and disrupted professional growth, resulting in attrition of research‐capable Aboriginal staff Research career pathways are unclear or inflexible, with poor clarity around qualification and work experience requirements (including how evaluation and continuous quality improvement experience in health service settings can map to research career capabilities) and related uncertainty regarding appropriate remuneration at different experience levels Research funding requirements for certain qualifications and track records that do not align with Aboriginal researchers' experience or perspective	Map Aboriginal and Torres Strait Islander research career pathways and establish tools, guidance and platforms to implement pathways in partnership with research institutes Target investments to ACCHSs and other remote health services to enable local Aboriginal staff to engage in research and ensure that remote knowledge generation includes a health industry perspective
3. Improving access to research education and training	Research project timelines frequently do not allow time or financial resources for researchers and community and service ‐based participants to access relevant training High staff turnover and limited leadership roles for Aboriginal people in remote locations disrupts access to mentorship	Enhance place‐based, flexible training programs and pathways to increase Aboriginal and broader remote‐based health workforce participation in research Connect remote researchers with virtual communities of practice that offer mentoring and support Improve broader health workforce recruitment, and retention and leadership development
4. Creating and leveraging partnerships	Limited infrastructure and high staff turnover in remote contexts limit research critical mass and result in personal relationships being the foundation of partnerships Person‐dependent partnerships can be fragile, risking the broader partnership as individuals leave While multiple partnerships and networks to improve remote capacity and capability are established and valuable, it is sometimes challenging to coordinate across these multiple groups and networks and avoid creating overlapping networks and new silos	Establish and strengthen research networks, especially those that connect new‐to‐research staff in services with established early and mid‐career and researchers Develop shared understandings of partners' research goals and initiate policy mechanisms to codify mutual expectations, roles, and responsibilities
5. Improving research funding, administration and management	Remote health services have minimal resources for research, and thus rely on project‐specific funding and limited staff time to conduct research that addresses their service evaluation and improvement needs Remote health service organisations are often already under‐staffed, with little if any time to access competitive funding sources. This increases the likelihood that remote research will be driven by external researchers' interests rather than local service priorities Even when research grants are secured, services face difficulties with research administration and management which can result in project delays	Advocate for longer term research investments to move beyond programmatic funding cycles, and that enable industry partners to lead and contribute to co‐design from the outset Establish interactive, centralised research portals that facilitate sharing data, guidance, tools and agreements between health services and researchers Share grants management support functions across organisations according to where this capability is best held, developed and shared Effective budgeting to account for the true cost of community and industry research engagement

Workshop actions highlight the importance of the broader national funding and policy environment for health systems research, particularly for primary health care (PHC) research. A priority action in Australia's PHC 10‐Year Plan 2022–2032 is to invest in research and evaluation to support locally delivered integrated care [13]. The Medical Research Future Fund (MRFF) and National Health and Medical Research Council (NHMRC) are the Government's main mechanisms for funding health systems research. In 2024, the Federal Government committed $100 million to PHC research over 10 years (2024–2025 to 2033–2034) under the MRFF Primary Health Research Plan [14]. This funding aims to support projects focussed on equity of access to health care by expanding the evidence base of service delivery and patient outcomes, emphasising knowledge translation [14]. Separate streams are available for applications with majority investigators based in regional, rural, and remote areas and for Indigenous health research. Yet, even with this increased targeted funding, competitive and programmatic funding models (i.e., funding for 2–5 year projects) limit the ability of remote researchers and partners to sustain research engagement [11]. Moreover, remote‐based researchers can easily become overburdened: investment in research hubs and networks to promote collaboration and skills sharing can help to overcome isolation and build critical mass in remote areas [5].

Previous funding schemes provide an example of how a relatively modest funding quantum can deliver substantial benefit in high‐need contexts. For instance, the Primary Health Care Research, Evaluation and Development (PHCRED) Program, which commenced in 2000 and was funded by Australia's Department of Health and Aged Care to 2011, provided funding to selected university departments (46% of which were focussed on rural and remote health), allowing rural and remote‐based and early and mid‐career researchers easier access to resources than the competitive funding accompanying other relevant schemes (such as NHMRC funding) [15]. The program supported 661 publications in 212 journals from 2006 to 2010—representing a substantial contribution to the body of PHC knowledge, steady research contributions from rural departments on issues relevant to their community (despite fewer resources than urban counterparts), and development of research capacity in rural and remote PHC settings [15]. A key legacy of PHCRED was the Modified Monash Model (MMM) developed by the PHCRED‐funded Centre of Research Excellence for Rural and Remote Primary Health Care. This fit‐for‐purpose classification system was adopted by the Australian Government as the basis for better targeting resources for health workforce programs, demonstrating how research evidence generated locally can improve health workforce policy [16].

Other schemes, including the NHMRC Centres of Research Excellence, have leveraged the foundational people and infrastructure built through PHCRED—indeed, several current remote health research leaders in CRESTRA were supported in developing their careers through the PHCRED‐enabled program. However, modern schemes lack a longer term national strategy to guide rural and remote health investment and primarily fund projects. Ultimately, the end of PHCRED resulted in the loss of dedicated, strategy‐driven funding for remote PHC research and research training. While the current multidisciplinary Rural Health Training Program (University Departments of Rural Health and Rural Clinical Schools) has supported research capacity, capability and output in rural health, [17] there remains a need for a cohesive rural health research funding source [18]. In all, compared with PHCRED, current funding schemes for rural and remote health systems research are more fragmented, competitive and project‐specific.

## 3 How Can We, as a Community of Remote Health Researchers, Enhance Knowledge Translation in Remote Australia to Address Persistent Health Disparities?

Workshop discussions and actions highlight the importance of enabling real‐world impacts from research: a key principle agreed by workshop participants was that *addressing local needs and priorities through research requires two‐way communication between researchers, service and community partners and a sustained commitment to ensuring that research delivers practical benefits* [9, 10]. We already know that producing new knowledge is not enough. A key barrier to improving remote health is ‘a failure to translate our current knowledge into policy’, and an ‘unwillingness amidst short‐term political cycles to make the necessary investments needed for longer term improvements amidst prevailing racist, metro‐centric and self‐interest agendas of the majority’ [2]. Yet, researchers partnering with remote services still sometimes assume that implementing research findings, conceptualised as being separate from services and practices, is a linear process that ends once the findings are published. These assumptions can result in missed opportunities to improve service models and policies that continually fail to improve remote health outcomes, [19] and may be enabled by research funding schemes that primarily incentivise academic (i.e., knowledge production) impacts rather than policy or practice impacts.

Participants in the workshop strongly emphasised the need to focus efforts on strengthening investment in Aboriginal and Torres Strait Islander researcher career pathways and leadership of research. This is fundamental to ongoing efforts to Close the Gap in health and life expectancy outcomes between Aboriginal and Torres Strait Islander peoples and non‐Indigenous Australians [20]. Involvement of Aboriginal and Torres Strait Islander researchers and community across all phases of research is known to enable impactful research in Aboriginal and Torres Strait Islander health, and in turn, improve health and wellbeing [21].

Proposed actions also highlight the critical role of ACCHSs in strengthening knowledge translation. ACCHSs embody community‐led approaches and are often engaged in best practice research and policy implementation. ACCHSs are often approached by research institutions and external researchers seeking to engage and partner in research. In turn, ACCHSs aspire to greater staffing, funding and administrative capacity to systematise ethical research practices, to empower and enable them to generate and use evidence to inform service programs, systems, policy and practice [10]. Involving practising clinical staff in remote services in research design, implementation and reporting of research in their services is also critical for evidence‐based practice [22]. Successful models of research capacity and capability building in health services, such as those established within the Central Australian Aboriginal Congress (Congress), can provide lessons for other organisations that are developing their research engagement (Box 1). However, there are gaps in our understanding of research engagement priorities in other services, such as smaller, very remote ACCHSs, that may be interested in different approaches and models of research engagement to improve healthcare practice and wellbeing locally.

BOX 1 **The Research Development Journey at Central Australian Aboriginal Congress.**

Health services interested in research engagement and impact can draw lessons from the research development journey at Central Australian Aboriginal Congress (Congress). Congress is the largest Aboriginal Community Controlled Health Service (ACCHS) in the Northern Territory, providing a comprehensive, holistic and culturally appropriate primary health care (PHC) service to Aboriginal people living in or visiting Mparntwe (Alice Springs) and across Central Australia, including in nine remote or very remote communities: Amoonguna, Ntaria, Wallace Rockhole, Ltyentye Apurte (Santa Teresa), Utju (Areyonga), Imanpa, Yulara, Kultukatjara (Docker River) and Mutitjulu. In 2024, Congress celebrated its 50th birthday, and over time has become a national leader in improving health outcomes for all Aboriginal people and a strong advocate for Closing the Gap in Aboriginal health disadvantage.

Congress' research journey commenced in the 1980s with the development of Congress Research Guidelines in 1982, which established key principles for respectful research conduct with remote Aboriginal communities, countering largely biomedical and colonial approaches to health research that were previously dominant. Congress' Chief Medical Officer, Dr. John Boffa, notes ‘these were among the first attempts by Aboriginal and Torres Strait Islander people to place ethical and cultural restrictions on how health research was carried out in their communities’ [23].

This set a foundation for impactful projects addressing local community needs. Early projects included Health Business/Settle Down Country Pmere Arlaltyewele (1983), a project that sought to understand how Aboriginal people think about health and illness; All That Rama Rama Mob (1988), a project that investigated Aboriginal people's perceptions of behaviour, leading to the establishment of Disability Services Central Australia; and On the Machine (1998), a project that illuminated the difficulties faced by renal patients, prompting the formation of a Renal Working Group and accommodation for dialysis patients in Alice Springs.

In 1997, Congress was a founding partner in the new Cooperative Research Centre (CRC) for Aboriginal and Tropical Health, led by Aboriginal leader Lowitja O'Donoghue (the CRC later evolved into the Lowitja Institute in 2010). The CRC produced critical guidance on conducting research in Indigenous health and supporting Indigenous researchers and delivered research with a focus on practical outcomes, while also strengthening Congress' research partnerships with Danila Dilba Health Service in Darwin, the NT Health Department and academic partners including Menzies School of Health Research (Menzies). Later, other key ACCHOs and partners, including Menzies, came together as the Researcherenye Wappayalawangka‐Central Australia Academic Health Science Centre (CAAHSN), which was nationally accredited as an NHMRC Research Translation Centre in 2014.

Additional impactful projects led by Congress since the 1990s include Grog Mob in 2008 that evaluated multidisciplinary alcohol management interventions with Aboriginal clients (leading to a three streams of care approach that now informs Congress' social and emotional wellbeing program), a Continuity of Care Model Evaluation in 2021, the Congress History Project from 2023 (investigating how Aboriginal health status has changed over time and the impact of Congress services), and a 2025 project with Menzies funded by the Medical Research Future Fund on optimising health system integration through innovative models of multidisciplinary primary care in the remote Aboriginal context.

With support from Menzies and other research institutions, Congress established a dedicated Research Section in 2016 to conduct internally‐driven research [23]. This built from Congress' sustained cross‐organisational partnerships, access to grants that enabled employment of embedded researchers (including a key Aboriginal research officer role in 2019), and recruitment of critical research support roles. A research manager role was also established to support and conduct research. This role continues as the administrative foundation for the Congress Research Unit, that now consists of 12 research positions on average (fluctuating depending on funding) and supports > 100 research projects that are led by Congress or conducted in partnership with multiple research institutes and university partners nation‐wide (some projects are at the knowledge translation stage). A key resource, Congress' Aremella Arratyenye‐ileme Doing it Right: A Guide for Health Researchers Working in Central Australia (2021), was developed by Congress in consultation with town‐based services and remote health boards to provide guidance to internal and external researchers on culturally safe research design and conduct with Aboriginal clients and communities, adapted from the values in the NHMRC's Keeping Research on Track Guide. The Guide articulates six core values to guide impactful research with Aboriginal peoples in Central Australia: Uphold Culture; Justice and Fairness; Sharing; Respect and Relationships; Responsibility; and Commitment.

A research subcommittee of the Congress Board assesses research requests against community needs and priorities and culturally appropriate methodologies, constituting a dedicated research governance structure and reflecting a commitment to data sovereignty principles. Once research proposals have been approved by the Congress Research Subcommittee, they are put to the Congress Board (and subsequently the Boards of other Congress‐auspiced services) for review. Research projects must attain approvals from all levels of governance to proceed.

Despite its strengths, Congress' research effort is limited by significant in‐kind expectation and project‐based funding, which inhibits essential cross‐project facilitation and community engagement outside of specific projects, limits knowledge translation activity and threatens continuity of the research effort. Congress, with its partners, is exploring opportunities to ensure that these efforts are sustained and optimised and enable real‐time feedback to services to improve health care quality and access in Central Australia.

Key data sources:

- Boffa, J and Ah Chee, D [24].
- Reflections from Congress‐based co‐authors Vahab Baghbanian and Sam Moore.

NHMRC‐accredited Research Translation Centres (RTCs) were established across Australia from 2017 to institutionalise knowledge translation in Australia's jurisdictions and regions [25]. Two in the Northern Territory, the Central Australian Academic Health Sciences Network (CAAHSN) and the Top End Aboriginal Health Research Alliance (TEAHRA), support stakeholder researcher networks and connect local academic institutions with health services to enable place‐based research and impact [10]. Workshop discussions highlight that there is widespread support and enthusiasm among remote service providers to build knowledge translation pathways through RTCs, which can involve supporting and encouraging community driven health research from the ground up [10]. CAAHSN is generating impact assessment tools (e.g., exploring and adapting the Framework to Assess the Impact from Translational Health Research (FAIT) model [26]) that reflect the remote Aboriginal context and is well placed to trial knowledge translation approaches that amplify service and community impacts from research.

**FIGURE 1:**
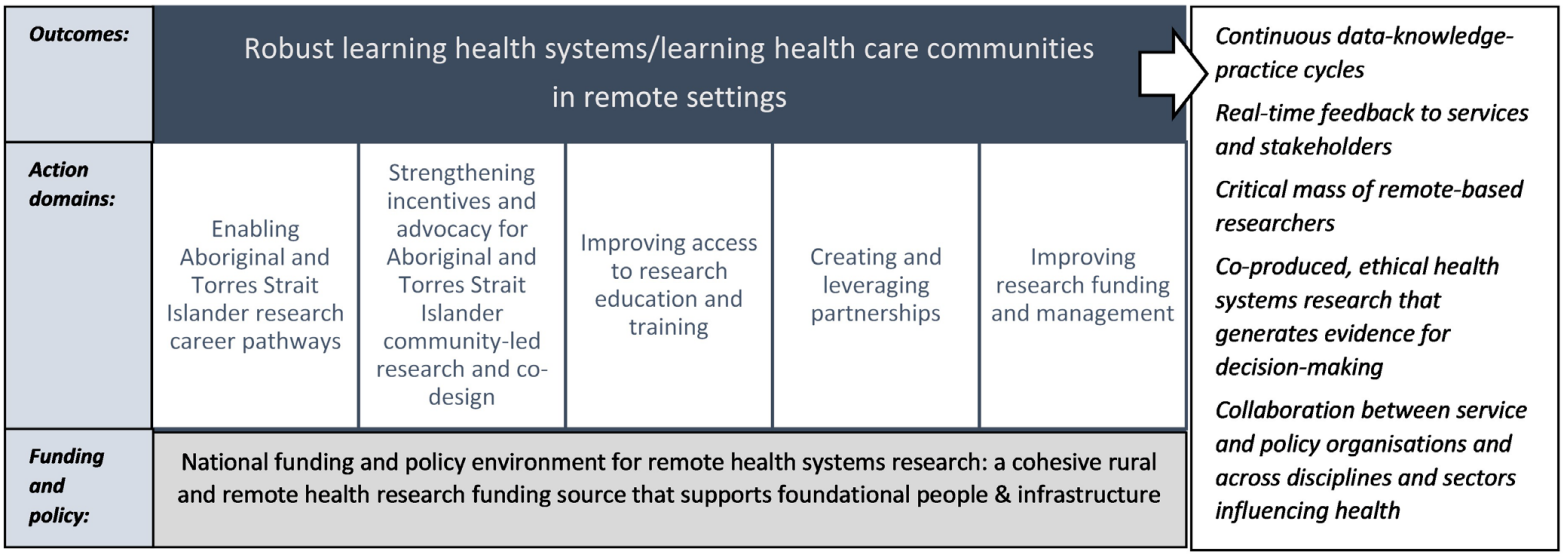
CRESTRA vision for strengthening remote research capacity, capability and knowledge translation.

Knowledge translation, knowledge mobilisation, and learning health systems are related concepts that are the focus of a vast and growing literature—though are often centred on urban hospital‐based settings. We recognise the importance of exploring conceptual nuance but anchor our reflections here on pragmatism and action. Our workshop suggests that learning health systems in remote Australia, enabled through RTCs and involving Aboriginal and Torres Strait Islander leadership, should facilitate continuous data‐knowledge‐practice cycles, giving real‐time feedback to services and stakeholders while helping to address the low critical mass of remote researchers and broader health workforce shortages. They should also enable co‐produced, ethical health systems research that generates evidence for decision‐making and enables collaboration between service and policy organisations and across disciplines and sectors influencing health. In this sense, a vision for learning health systems in remote Australia might more closely resemble ‘learning health care communities’ (LHCCs), which emphasise co‐development of solutions to complex health challenges and a focus on public health, social and environmental determinants of health, and community partnerships [27] – though downplaying ‘care’ and accentuating ‘systems’ in framing these efforts might be important [28]. We summarise our vision for strengthening remote research capacity, capability and knowledge translation in Figure 1, which encompasses the five action domains discussed in the workshop and learning health system outcomes, underpinned by a cohesive policy and funding model for remote health systems research.

## 4 Conclusion

Barriers to sustained remote health research engagement have been recognised for decades, [29] yet feedback from remote service providers and researchers suggest that there are still gaps in resourcing and support, despite exemplary pockets of innovation. Our workshop, held in Mparntwe in 2024, highlights that the time to act is now: remote‐based researchers and service providers are seeking support from national research funders and policymakers to strengthen remote health systems research and knowledge translation to address persistent health inequities. Action must include creating funding schemes that provide for cohesive, systematic, and sustained investment in remote‐based health systems research partnerships that involve industry partners, especially ACCHSs, to contribute to Closing the Gap in health outcomes. Partnerships must respond directly to community and service provider priorities, create Aboriginal and Torres researcher career pathways, and leverage respective partners' institutional strengths to create learning health systems. Critically, actions also require strengthening overall remote health workforce recruitment and retention to improve foundational workforce stability.

## Author Contributions

**Alexandra Edelman:** conceptualization, writing – review and editing, writing – original draft. **Sam Moore:** conceptualization, writing – review and editing, writing – original draft. **Karrina DeMasi:** conceptualization, writing – review and editing. **Donna‐Maree Stephens:** conceptualization, writing – review and editing. **Vahab Baghbanian:** writing – review and editing. **Chris Perry:** conceptualization, writing – review and editing. **Deborah Russell:** writing – review and editing. **Kalinda Griffiths:** writing – review and editing. **Leisa McCarthy:** writing – review and editing. **John Humphreys:** writing – review and editing, supervision. **John Wakerman:** writing – review and editing, supervision.

## Funding

This work was supported by National Health and Medical Research Council, GA294950.

## Disclosure

The workshop discussed in this manuscript was supported by the NHMRC Centre of Research Excellence for Strengthening Health Systems in Remote Australia (CRESTRA). The authors are researchers, policymakers, health administrators and community members with extensive professional and lived experience in remote health systems and place‐based research. The authors are co‐researchers in CRESTRA. Karrina DeMasi, Donna Stephens, Kalinda Griffiths and Leisa McCarthy are Aboriginal research leaders with extensive experience in remote health systems and Indigenous research capacity building. Sam Moore and Vahab Baghbanian (Central Australian Aboriginal Congress) and Chris Perry (Aboriginal Medical Services Alliance Northern Territory) work in the Aboriginal Community Controlled Health Service sector. Alexandra Edelman, Deborah Russell, John Humphreys and John Wakerman are health systems researchers. Members of the authorship team convened the workshop on remote research capacity building in Mparntwe in 2024 and are leading the further development, implementation, and facilitation of strategic actions from the workshop.

## Conflicts of Interest

The authors declare no conflicts of interest.

## Data Availability

Data sharing not applicable to this article as no datasets were generated or analysed during the current study.
